# CFTR Deletion in Mouse Testis Induces VDAC1 Mediated Inflammatory Pathway Critical for Spermatogenesis

**DOI:** 10.1371/journal.pone.0158994

**Published:** 2016-08-02

**Authors:** Chen Yan, Qin Lang, Liao Huijuan, Xie Jiang, Yang Ming, Sun Huaqin, Xu Wenming

**Affiliations:** 1 Department of Obstetrics and Gynecology, West China Second University Hospital, Sichuan University, Chengdu 610041, Sichuan, China; 2 Key Laboratory of Birth Defects and Related disease of Women and Children, Ministry of Education (Sichuan University), West China Second University Hospital, Sichuan University, Chengdu 610041, Sichuan, China; 3 SCU-CUHK Joint Laboratory for Reproductive Medicine, West China Second University Hospital, Sichuan University, Chengdu 610041, Sichuan, China; 4 Third People’s Hospital of Chengdu, the Second Affiliated Hospital of Chengdu, Chongqing Medical University, Chengdu 610031, Sichuan, China; Nanjing Medical University, CHINA

## Abstract

Cystic fibrosis is the most common genetic disease among Caucasians and affects tissues including lung, pancreas and reproductive tracts. It has been shown that Endoplasmic Reticulum (ER) stress and heat shock response are two major deregulated functional modules related to CFTR dysfunction. To identify the impact of CFTR deletion during spermatogenesis, we examined the expression of spermiogenesis-related genes in the testis of CFTR mutant mice (CF mice). We confirmed expression changes of MSY2, a germ cell specific RNA binding protein, resulting from deletion of CFTR in testis. Furthermore, real time PCR and Western blot results showed that an inflammatory response was activated in CF mice testis, as reflected by the altered expression of cytokines. We demonstrate for the first time that expression of MSY2 is decreased in CF mice. Our results suggest that CFTR deletion in testis influences inflammatory responses and these features are likely to be due to the unique environment of the seminiferous tubule during the spermatogenesis process. The current study also suggests avenues to understand the pathophysiology of CFTR during spermatogenesis and provides targets for the possible treatment of CFTR-related infertility.

## Introduction

Cystic fibrosis is a genetic disease caused by the mutation of Cystic fibrosis transmembrane conductance regulator (CFTR). The link between CFTR mutation and congenital bilateral absence of the vas deferens (CBAVD) has long been established by clinical observation as well as animal studies [1, 2]. Our previous study and others have found that CFTR is expressed in mouse and human sperm and play important roles in sperm capacitation, however, whether CFTR dysfunction is related to spermatogenesis defects is still unclear [3–5]. European studies have shown that azoospermia patients have significantly higher 5T mutations compared with oligospermia patients, suggesting CFTR mutations could be related to defective spermatogenesis in humans [6, 7]. On the other hand it is known that people from east Asia have a very low incidence of the CFTR mutation related to classic CF disease [8]. In studies conducted in Chinese populations, the 5T mutation frequency is significantly higher in both CBAVD and azoospermia patients, compared to fertile controls. In the same study, further meta-analysis has confirmed the results, showing that the CFTR mutation is associated with azoospermia [9].

Despite the role of CFTR in CBAVD having been well established, its role in normal spermatogenesis remains unclear and CF mice provide a good model to study this. We have recently shown that CFTR deletion in CF mice causes spermatogenesis defects with compromised CREB activation in Sertoli cells. The mechanism is possibly related to defective HCO_3_^-^ transport and sAC mediated cAMP production [10]. It should be noted that CFTR is expressed in both germ and Sertoli cells [11], indicating that CFTR defects may affect different cell types and several stages during spermatogenesis.

Proteomics is a powerful technique to delineate the function of CFTR and proteomics. Together with the interactome in lung and pancreas tissues, recent studies have identified several proteins modulated by CFTR as potential drug targets [12–15]. One of the major findings in these studies is the heat shock and ER-unfolded protein responses (UPR response). These constitute major CFTR-related pathways contributing to the phenotype of the pathophysiology [16, 17], among which GRP78 has emerged as a major heat shock protein involved in CFTR transport [18]. Although these studies have provided insights into cystic fibrosis pathology from high-throughput approaches, it should be noted that the testis, which produces sperm, has a unique environment for CFTR function. Spermatogenesis takes place at 33°C instead of 37°C, which is a favorable environment for the maturation of CFTR, and is unique compared with any other system [19]. We hypothized that CFTR is a central regulator of spermatogenesis both in germ and Sertoli cells and therefore, CFTR defects could affect multiple aspects of spermatogenesis. To decipher the function of CFTR in spermatogenesis, first we studied the expression of MSY2, an RNA binding protein essential for spermatogenesis in CF mice testis [20]. We also examined the expression of major heat shock proteins involved in the UPR pathway in CFmice testis. Finally, whether CFTR deletion in testis could lead to increased oxidative stress levels, leading to altered expression of cytokines were examined in CF mice model.

## Material and Methods

### Testis tissue, protein extraction and Western blots

The *cftrtm1Unc* (S489X) mice were ordered from Jackson labs and maintained in the LASEC of CUHK. Manual cervical dislocation was used for euthanasia of the mice. The animal experiments were all approved by the Animal Research Ethics Committee of the University (Ref. No: 04/025/ERG; CUHK 4360/04M). Cell lysates were prepared using RIPA lysis buffer (10 mM Tris, 0.15 M NaCl, 2 mM PMSF, 2 mM EDTA, 2 mM N-ethylmaleimide, 1% NP-40 [v/v], 10% glycerol [v/v], pH 7.4 at 22°C). Whole cell and testis lysates were prepared as described [21]. For each sample, about 100 μg protein in 30 μl were resolved by SDS-PAGE under reducing conditions. Western blotting and detection of target proteins were performed as previously described [10].

### Real time PCR

Total testis RNA was prepared from frozen tissues with Trizol reagents (Life Technologies, USA). A total of 500ng RNA was used for reverse transcription, which was carried out using the PrimeScript^™^ RT reagent Kit (Takara, Dalian, China) and was performed in accordance with manufacturer instructions for M-MLV reverse transcriptase (Life Technologies, USA). Quantitative real-time PCR (qRT–PCR) analysis was performed using an Applied Biosystems 7500 detection system (Life Technologies, USA). For the detection of cDNA, the experiment was carried out following the instructions of the SYBR^®^ select master mix (Life Technologies, USA). The specificity of primers and probes used for qPCR were validated with melting curve analysis, in which only one peak was observed. Primer sequences are shown in S1 Table.

### Co-IP Protocol in testis and cell culture

Co-immunoprecipitation (Co-IP) was performed as previously described [21]. Briefly, lysates of testes, seminiferous tubules, and Sertoli cells were prepared using the lysis buffer as described above. Equal amounts of proteins (400 μg) were pretreated with normal rabbit serum (1:150) for 3 hours at room temperature. Thereafter, 20 μl Protein A/G PLUS-agarose (Santa Cruz Biotechnology) was added and incubated at room temperature for an additional 3 hours to precipitate serum proteins that would non-specifically interact with IgG. After removal of the agarose beads by centrifugation at 1000 × *g* for 5 min, supernatants from each sample were incubated overnight with the corresponding antibody (1:150) at room temperature with agitation on a rotator at 24 rpm (Glas-Col, Glass Tech Supplies Inc., Fullerton, CA). Subsequent incubation was completed at room temperature with the addition of 20 μl of Protein A/G PLUS-agarose suspension for 4 hours to recover the immunocomplexes. Immunoprecipitates were washed four times with the lysis buffer by resuspension and centrifugation (1000 × *g*, 5 min each). Samples were denatured in SDS sample buffer and resolved by SDS-PAGE under reducing conditions.

### ATP measurements in testis

Each testicle was homogenized in a solution containing 3% trichloroacetic acid (TCA)-2 mM EDTA, and homogenized in a dounce homogenizer. After incubating for 15 minutes at room temperature, the lysates were centrifugated at 16,000 g for 10 minutes and the ATP content in the supernatants measured using a Bioluminescent ATP Assay kit (Sigma). The assay mixture containing 20 μl of cell extract and 100 μl of ATP assay mix (FL-AAM) was diluted 25-fold in adenosine 5′triphosphate (ATP) assay mix dilution buffer (FL-AAB) according to the instructions from the suppliers. Measurements of chemiluminescence of the luciferin-luciferase reaction was made in a Thermo Varioskan Flash spectrum. The standard samples were diluted 10 times with 10^−9^ to 10^−5^ buffer and the results were converted to gram/ml/mg tissue.

### Immunohistochemical staining

Immunohistochemical staining was performed according to standard methods using an immunohistochemistry kit (Histostain TM-Plus Kits, Rabbit. Number: SP-9001; Beijing Zhongshan Biotechnology Co., Ltd) with minor revisions. Briefly, the tissues were embedded in paraffin blocks. Next, 5-mm sections were sequentially cut and mounted onto gelatin-coated slides. Slides with tissues were dried overnight at 37°C, then deparaffinized in xylene and rehydrated through a series of graded ethanol. To retrieve the epitope, slides were immersed in citrate buffer at pH 6.0 in 120°C water for 20 minutes. The slides were cooled to room temperature for 20 minutes and then incubated in H_2_O_2_ (0.3%) at room temperature for a further 20 minutes. After serum blocking, the slides were incubated with nitrotyrosine antibody (l: 100, Millipore, Billerica, MA) at 4°C overnight. PBS instead of primary antibody was used as the blank control and the placenta from preeclampsia tissue was used as a positive control. Anti-rabbit secondary antibody and DAB (DAB Horseradish Peroxidase Color Development Kit) reagents were added from the Histostain TM-plus kit (Beijing Zhongshan Biotechnology Co., Ltd, Beijing, China). Immunohistochemical results were analyzed using a light microscope (TI-U, Nikon CLEIPSE, NIKON, Japan), which was equipped with a camera (SPOT Flex TM Camera, Diagnostic Instrument, USA). The image analysis was performed using SPOT advance software.

### Statistical analysis

All the data were presented as mean+/-SEM unless specified. The Graph Prism 5 statistical package was used for statistical analysis. The F-test of two-sample homogeneity of variance was used to analyze the homogeneity of the samples. Students t-test and 1-way ANOVA with a post-hoc test were used for the comparison between two groups and three groups (WT, heterozygous and homozygous CF mice). A probability (P) <0.05 was considered statistically significant.

## Result

### MSY2 and the downstream genes were altered in CF tissue

**Fig 1:**
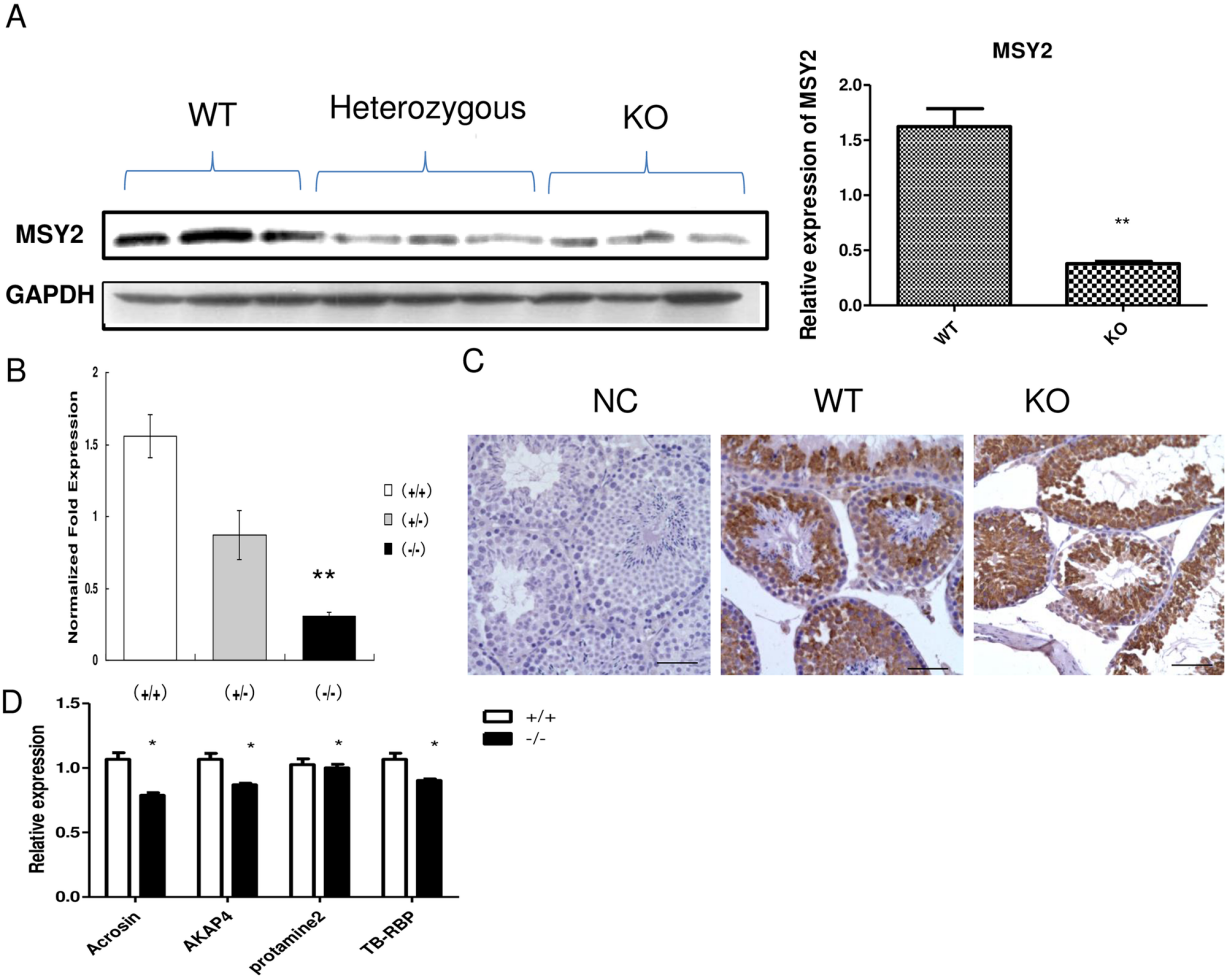
The expression of MSY2, a germ cell specific gene was significantly reduced upon CFTR deletion. (**A**) Western blot shows that significantly reduced MSY2 gene expression were detected in Cftr(-/-) mice testis. Right panel is the statistic result. (**B**) Real time PCR confirmed that MSY2 transcript is also reduced in Cftr(-/-) mice testis compared with wide type mice (** means P<0.01,N = 3). (**C**) Immunohistochemistry indicate that MSY2 is localized in spermatocyte and spermatid cells. (**D**) Real time PCR shows that the expression of Msy2 regulated genes, including Acrosin, AKAP4, Cyclin A1 and TB-RBP gene were significantly reduced in Cftr(-/-) mice testis. (* means P<0.05;** means P<0.01,N = 3).

MSY2 is an RNA binding protein essential for spermiogenesis [20]. To check whether CFTR deletion could have an impact on spermiogenesis in germ cells, we used MSY2 as a marker to determine whether MSY2 expression was affected in CF mice. Our results showed that the expression of MSY2, the key RNA binding protein, is significantly reduced in CF mice (Fig 1A). Real time PCR also shows that MSY2 expression is significantly inhibited at the RNA level, indicating that MSY2 expression regulation is at the transcription level (Fig 1B). Immunohistochemistry staining shows that MSY2 is highly expressed in germ cells, with spermatid cells showing the strongest expression, and reduced expression of MSY2 is observed in CF mice testis (Fig 1C). In accordance with MSY2-mediated regulation, real-time PCR indicated that the expression of MSY2 regulated genes, including acrosin, AKAP4 and TB-RBP was also significantly reduced (Fig 1D).

### VDAC1 mediated inflammatory pathways in CF mice testis

**Fig 2:**
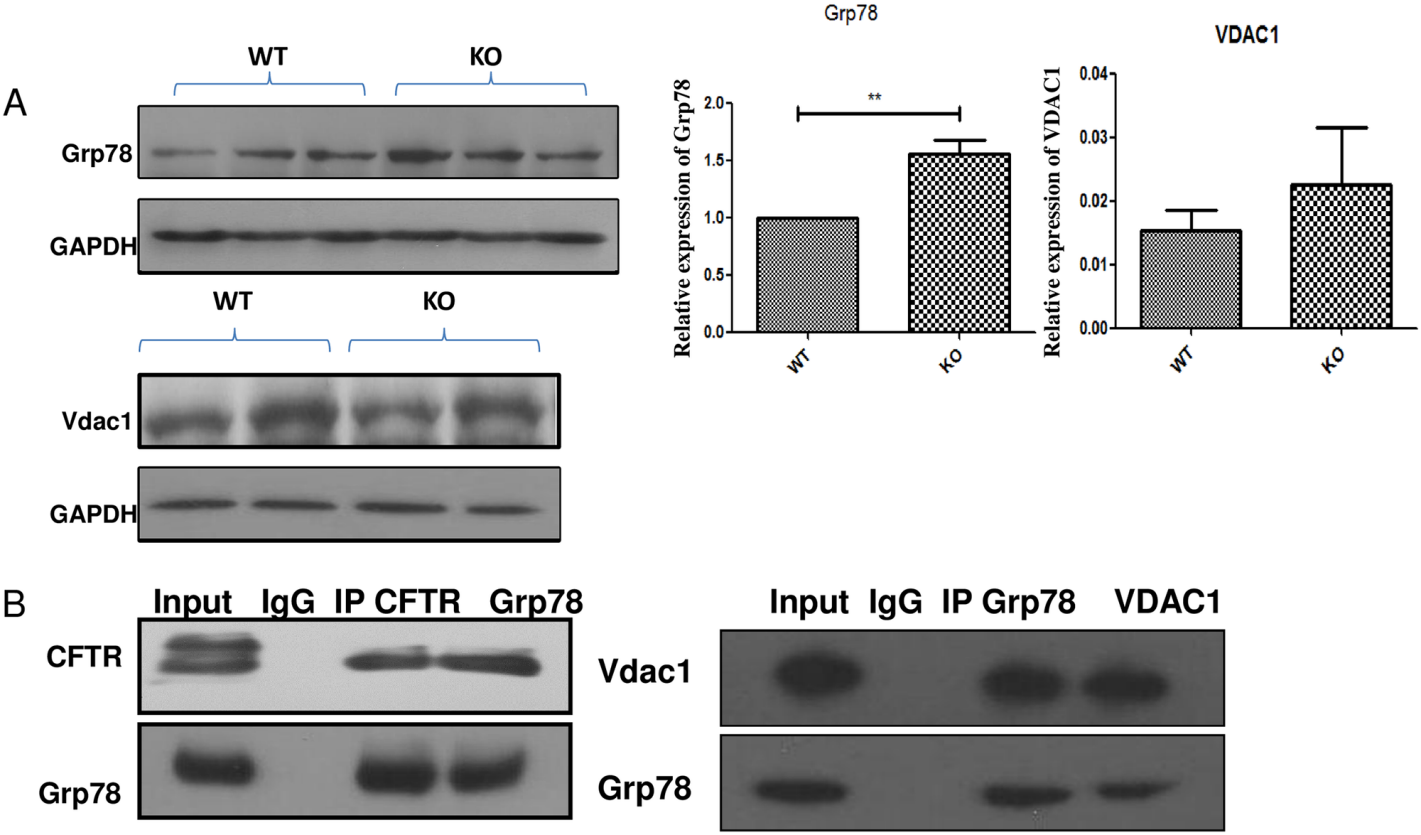
ER and Mitochondrial protein dysfunction may contribute to spermatogenesis defects in CF mice. (**A**) The expression of heat shock protein, ER and mitochondrial genes, including Grp78 and VDAC1 were increased in CFTR mutant mice, right panel is the statistic result. (**B**) Upper panel: Co-IP result confirm that Grp78 could interact with immature band of CFTR in mice testis. Down panel: VDAC1 and Grp78 could interact with each other in mice testis.

**Fig 3:**
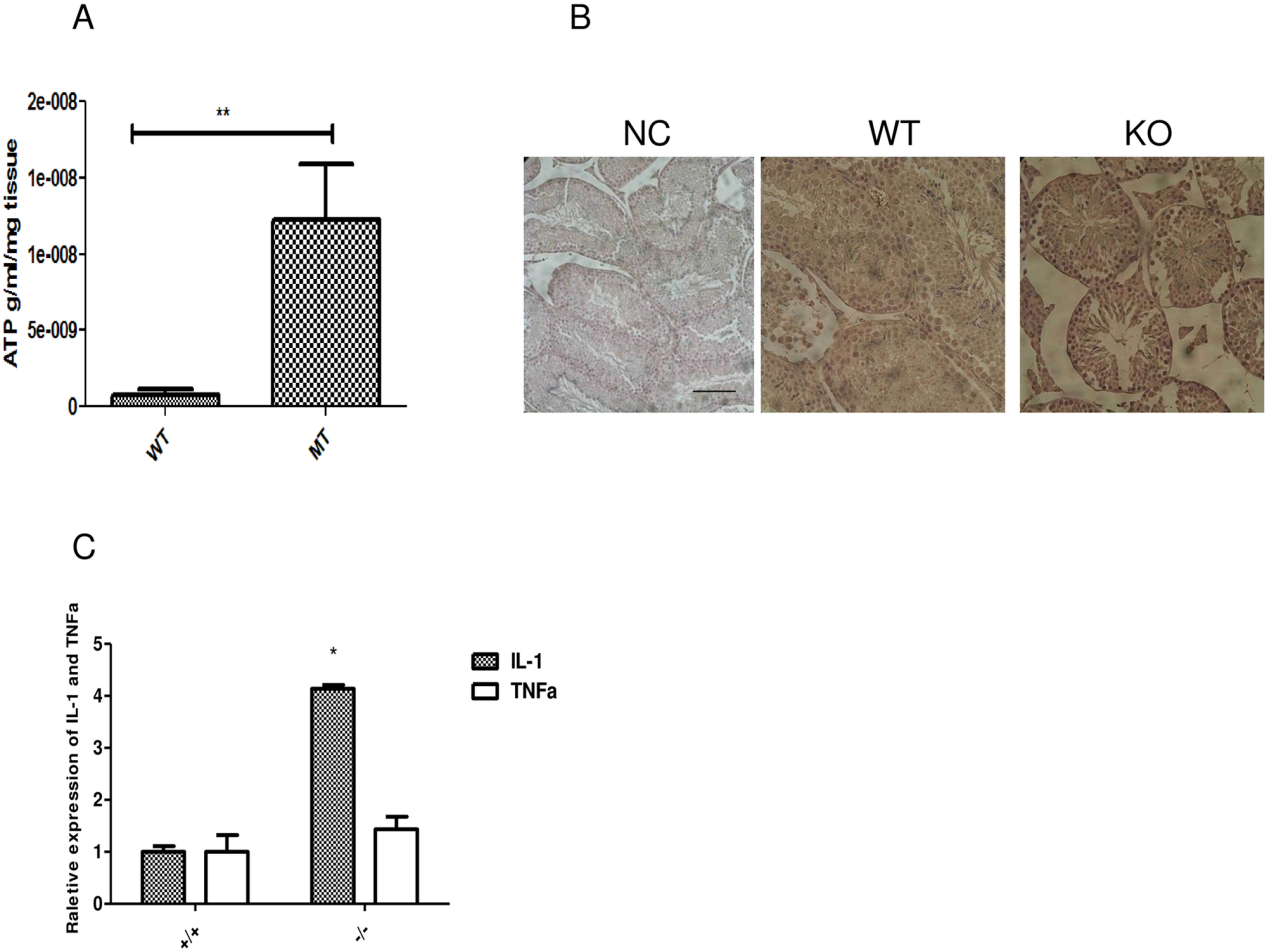
(**A**) ATP measurement shows that Cftr(-/-) mice has significantly increased ATP production compared with WT mice testis. (**B**) Immunohistochemistry staining of nitrotyrosine, an oxidative stress marker in testis shows that mutant mice has significant increased signal in seminiferous tubule. (**C**) cytokine profile further confirm that IL-1 level is significantly increased, while TNF-alpha expression shows no significant change in Cftr(-/-) mice testis.

Heat shock proteins are the major contributors responsible for normal CFTR folding to the cell surface, and the deregulated expression of chaperones is involved in the misfolding of the CFTR protein. We therefore checked the expression of major heat shock proteins using western blots in CF mutant mice testis. Recent study showed that Grp78 is an ER stress signal that is related to mitochondrion function, possibly through mediation of VDAC1 [22]. Mitochondrial function defects may be important for the underlying mechanism of CF mutation. Therefore, it is possible that the heat shock protein GRP78(Bip) mediated VDAC1 function is altered in CF mice testis. Western blot results show that a significant change in Grp78 expression was detected in CF mutant mice (Fig 2A). VDAC1 expression in mutant mice testis is also slightly increased compared with WT control, but this did not achieve statistical significance (Fig 2A). To further check whether Grp78 could interact with CFTR directly, we then used a Co-IP experiment to confirm that Grp78 can interact with VDAC1 and CFTR in testis. The co-IP experiment showed that CFTR can bind to Grp78 and VDAC1 could also bind to Grp78 (Fig 2B). Thus, the CFTR mutation could lead to an altered heat shock response and VDAC1 expression. VDAC1 plays critical roles in ATP production, which could induce oxidative stress [23]. We therefore measured ATP production in the testis. Unexpectedly we found that the ATP concentration in mutant mice was significantly higher in mutant mice than in wild type controls, indicating that the CF testis has higher ATP synthesis compared with WT mice (Fig 3A). To further confirm that the higher ATP concentration was related to ROS production, we used nitrotyrosine staining to determine oxidative stress levels in CF mutant testis sections. Results showed slight increases in nitrotyrosine staining in the CF testis sections (Fig 3B), indicating that the CFTR mutation leads to oxidative stress during spermatogenesis.

It is well known that the CFTR mutation affects cytokine secretion in lung and other tissues. Interestingly, we have also found that IL-1α expression is significantly up-regulated in CF testis (Fig 3C), indicating that the up-regulated expression of heat shock protein and over-activated VDAC1 mediated pathway could be responsible for the pro-inflammatory state in the CF testis.

### CFTR could regulate NF-κB directly in germ cell culture

**Fig 4:**
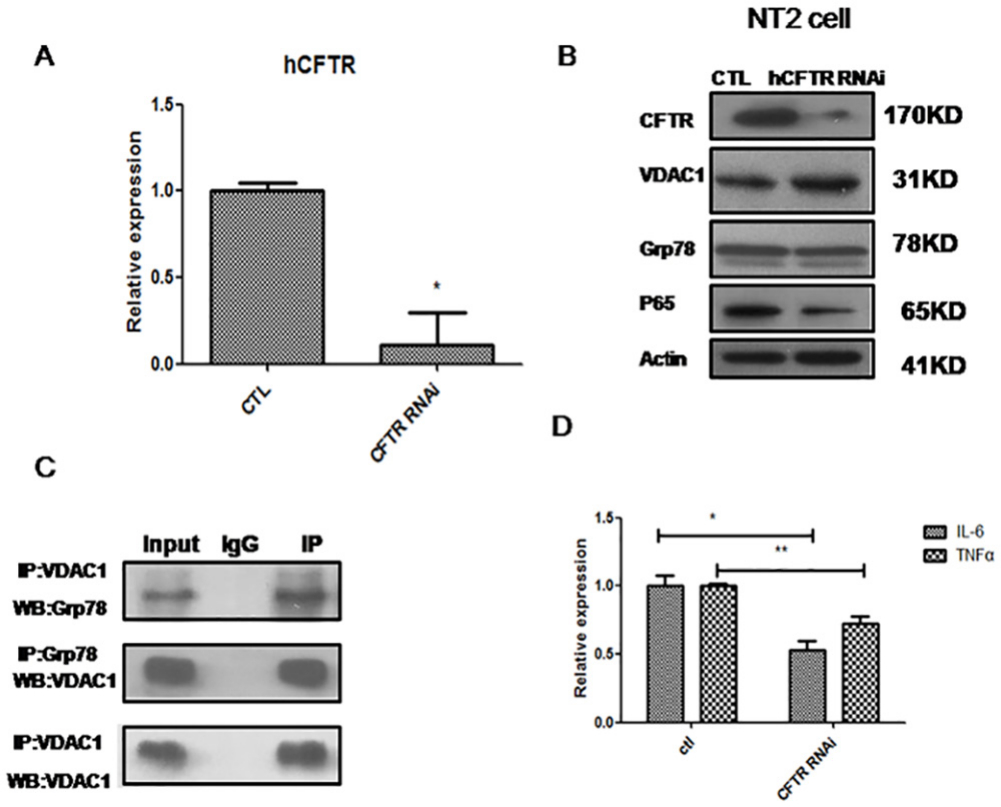
In vitro CFTR knockdown inhibit NF-κB and down-stream targets in NT2 cell model. (**A**) Real-time PCR shows that CFTR RNAi could inhibit CFTR transcription successfully, and (**B**) Knockdown of CFTR increase VDAC1 expression,while P65 expression was inhibited significanltly. (**C**) Co-IP data shows that Grp78 could interact with VDAC1. The upper two panels are the recipral immunoprecipitation result. The down panel is the Western blot result detecting VDAC1 after immunoprecipitated with VDAC1 antibody. (**D**) CFTR RNAi could inhibit IL-6 and TNF-alpha expression. (* means P<0.05, ** means P<0.01,N = 3).

It has been shown that CFTR can regulate Grp78 and NF-κB during embryo development and during cancer initiation. We chose NT2, a human germ cell tumor cell line to see whether knock down of CFTR affects the VDAC1 mediated pathway. Our results showed that, knock down of CFTR inhibits P65 expression, while the expression of VDAC1 was increased (Fig 4A and 4B). Furthermore, Co-IP experiment confirmed the direct interaction of VDAC1 and Grp78 in NT-2 germ cells (Fig 4C). Interestingly, the expression of two major cytokines, IL-6 and TNF-α was significantly reduced, indicating that CFTR could modulate cytokine secretion directly in germ cells (Fig 4D). To further confirm whether CFTR could affect NF-κB pathway in mice germ cell, we used GC-2 cell line, a mice spermatid derived germ cell line, and the result shows that knock down of CFTR also reduced P65 expression compared with control siRNA (S1 Fig), therefore our result support the notion that CFTR could regulate NF-κB pathway directly in germ cell.

## Discussion

There are several key findings for the function of CFTR during spermatogenesis in our current study. First, a CFTR defect in the testis affects MSY2 expression. Second, it also affects GRP78 as well as VDAC1 expression in testis and germ cells. Third, this CFTR defect may be related to the overproduction of ATP and ROS production, leading to altered expression of cytokines. Our results show that CFTR is a central regulator of spermatogenesis both in germ and Sertoli cells and therefore, CFTR defects will affect multiple aspects of spermatogenesis [3, 24].

MSY2 is a RNA/DNA binding protein, which has been shown to be critical for mRNA translation during spermatogenesis [20]. Deletion of MSY2 causes mRNA translation delay and it has also been shown that MSY2 binds a class of MSY2-binding miRNA [20], although the exact roles of these miRNAs in spermatogenesis remains undefined. Our data indicate that deletion of CFTR affects MSY2 and down-stream target genes. To our knowledge, this is the first study to demonstrate that CFTR deletion affect MSY2 expression. A recent study has shown that the mutation and polymorphism of YBX2, the human homologue of MSY2, are associated with azoospermia [25]. Therefore, the deletion of CFTR could also lead to azoospermia through the MSY2 mediated pathway. One unanswered question in the current study is the mechanism whereby the CFTRmutation affects MSY2 expression. Notably MSY2 belongs to a cold activated family of genes and one possibility is that the defective CFTR could affect miRNA production and miRNA could affect MSY2 synthesis in testis[26]. In support of the hypothesis, we have also shown that CFTR can affect miRNA production in testis [27]. Further experiments are warranted to investigate the exact mechanism for how CFTR deletion affect MSY2 expression and whether miRNA is involved in the regulation.

Similar to lung and in vitro systems, heat shock proteins (Hsp) play important roles for CFTR transport and maturation [28]. Of the several heat shock proteins affected by CFTR targets, Grp78 (Bip) is noteworthy since it has been shown to play important roles in other systems [16]. An Hsp inhibitor has recently been approved by the FDA for cancer treatment [29]. Whether these drugs could be used to treat CFTR dysfunction should be further explored. For example, it has been shown that Hsp90b and Grp78 (Bip) play an essential role in CFTR interactions [17]. Our study shows that Grp78 (Bip) affects mitochondrial function by linking with the mitochondrial protein VDAC1. Over-active ATP production indicates that altered energy metabolism is one of the key features affected by the CFTR mutation, and Grp78 and VDAC1 may play important roles in the observed mitochondrial dysregulation.

CFTR dysfunction is also related to pro-inflammatory status [30, 31]. In the current study, we found that CFTR mutant mice show increased expression of NF-κB and cytokines such as IL-1. The increased expression of NF-κB is responsible for the deregulated expression of cytokines. Interestingly, contrary to the *in-vivo* data showing that the CFTR mutation induced significantly increased expression of cytokines such as IL-1, our NT-2 germ cell model shows that knockdown of CFTR increased VDAC1 expression and decreased NF-κB expression, as well as diminished IL-6 and TNF-a expression. There are several possible explanations for these different results. Firstly, it should be noted that CFTR is expressed in both germ cells and Sertoli cell [3, 32]. The complex interactions of germ cells and Sertoli cells may cause different molecular changes between mouse models and human cells. Secondly, NT-2 is a testis embryonic carcinoma cell line, which may not reflect the normal germ cell condition *in-vivo*. Thirdly, the mouse CFTR and human CFTR may have intrinsic differences in terms of function and related pathways [33], which indicates the need for caution when directly correlating findings from mouse models with those in humans. Nevertheless, our results indicate that pro-inflammatory status represents a dominant feature affected by the CFTR mutation

**Fig 5:**
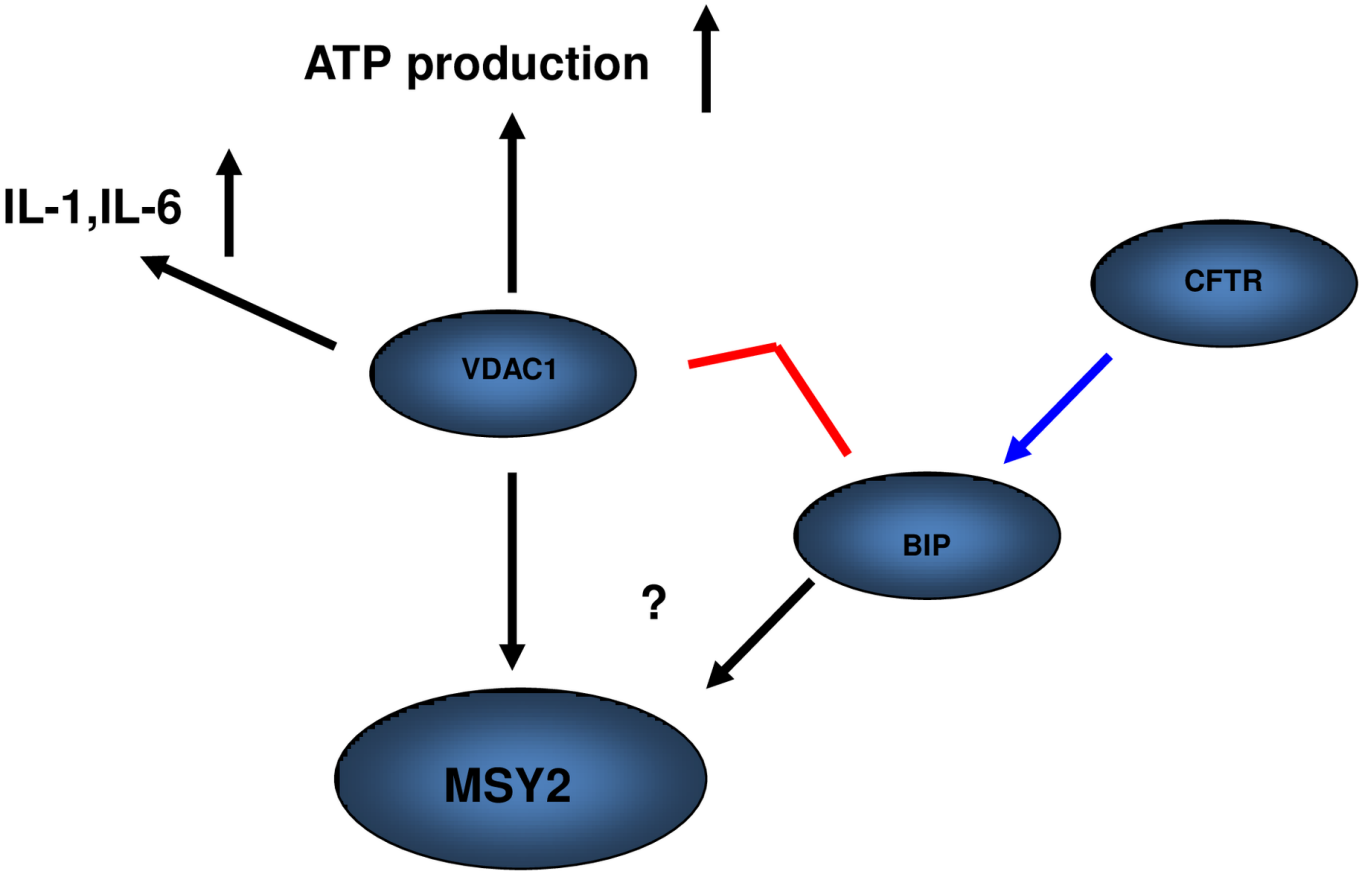
Proposed model to explain the specific function of CFTR in germ cell development. In the current model, CFTR plays a critical role in spermatogenesis through regulation of heat shock proteins and related pathways. We propose that CFTR mutations lead to an over-activated heat shock response, and reduced Grp78 and VDAC1 interactions to initiate the cascade leading to altered energy metabolism and ROS production. Furthermore, over-activated NF-κB activates the pro-inflammatory response and related interleukin increase. Finally, the key RNA binding protein MSY2 expression was significantly reduced, indicating that CFTR affect spermiogenesis through regulation of MSY2 expression.

We propose a model to explain the involvement of CFTR in spermatogenesis (Fig 5). In the proposed model, CFTR plays a critical role in spermatogenesis through regulation of heat shock proteins and related pathways. We propose that CFTR mutations lead to an over-activated heat shock response, and reduced Grp78 and VDAC1 interactions to initiate the cascade leading to altered energy metabolism and ROS production. Furthermore, stimulated NF-κB activates the pro-inflammatory response and related interleukin increase. Finally, the key RNA binding protein MSY2 expression was significantly reduced, indicating that CFTR affect spermiogenesis through regulation of MSY2 expression.

Whether the CFTR mutation/haplotypes affect human spermatogenesis is a long debated question with conflicting results from different ethnic groups [34]. It is well established that Caucasians are more likely to develop classic CFTR disease and show a higher mutation rate, such as 508 mutation, compared with Chinese and other east Asian populations [3]. A recent study showed that the 5T haplotype is relatively more common compared with fertile controls, indicating that same mutation of CFTR may be shared with different ethnicities [6, 9, 35, 36]. Several studies have shown that IVF patients have more 5T mutations; therefore it has been recommended that a CFTR mutation screen should be conducted in IVF clinics as routine practice. Clearly, understanding the mutation profiles in CF patients could contribute to the further design of drugs targeting CFTR in male infertility[37].

In conclusion, our current study has identified the key proteins and related pathways affected by the CFTR mutation in germ cells, and suggests the need for further investigation of how CFTR dysfunction affects spermatogenesis and its underlying mechanism.

## Supporting Information

S1 Fig In vitro CFTR knockdown inhibit NF-κB in GC-2 germ cell model. (**A**) Real-time PCR shows that CFTR RNAi could inhibit CFTR transcription successfully, and (**B**) Knockdown of CFTR increase Grp78 expression, while P65 expression was inhibited significanltly.
(TIF)

S1 Table The primer sequences used in the current study. (XLS)
