# Supplementary figures and images for: CFTR Deletion in Mouse Testis Induces VDAC1 Mediated Inflammatory Pathway Critical for Spermatogenesis
Source: PLoS One. 2016 Aug 2;11(8):e0158994. doi: 10.1371/journal.pone.0158994 (PMC4970767; doi:10.1371/journal.pone.0158994)

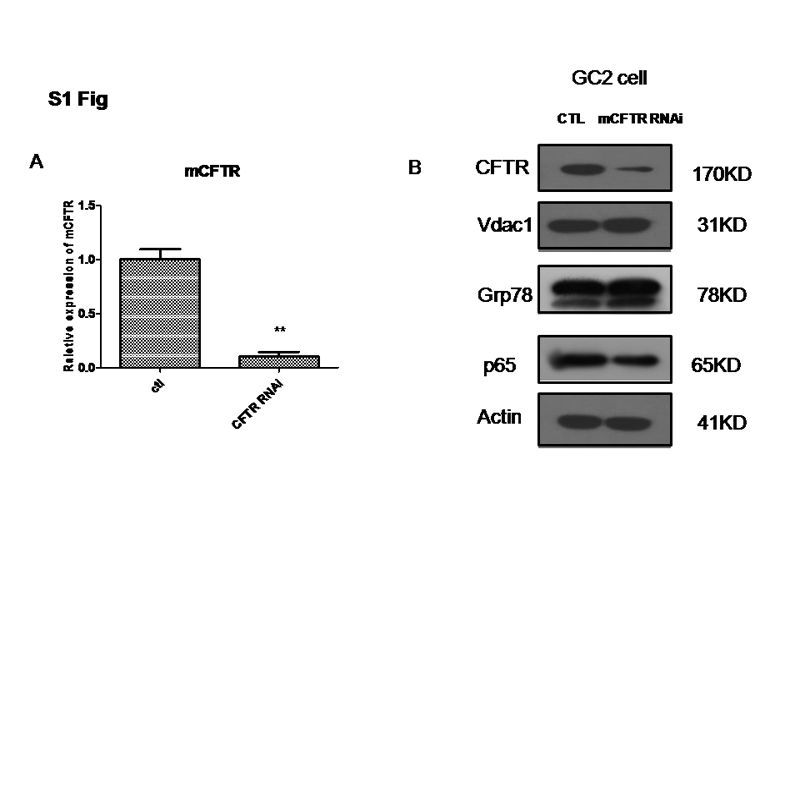

Supplement: S1 Fig — (A) Real-time PCR shows that CFTR RNAi could inhibit CFTR transcription successfully, and (B) Knockdown of CFTR increase Grp78 expression, while P65 expression was inhibited significanltly. (TIF) [file pone.0158994.s001.tif]
